# Disentangling the intersection of inequities with health and malaria exposure: key lessons from rural communities in Northern Borneo

**DOI:** 10.1186/s12936-023-04750-9

**Published:** 2023-11-09

**Authors:** Nurul Athirah Naserrudin, Pauline Yong Pau Lin, April Monroe, Sara Elizabeth Baumann, Bipin Adhikari, Anna Cohen Miller, Shigeharu Sato, Kimberly M. Fornace, Richard Culleton, Phaik Yeong Cheah, Rozita Hod, Mohammad Saffree Jeffree, Kamruddin Ahmed, Mohd Rohaizat Hassan

**Affiliations:** 1 Department of Public Health Medicine, Faculty of Medicine, Universiti Kebangsaan Malaysia, 56000 Kuala Lumpur, Malaysia; 2 Borneo Medical and Health Research Centre, Faculty of Medicine and Health Sciences, Universiti Malaysia Sabah, 88400 Kota Kinabalu, Sabah Malaysia; 3 Sabah State Health Department, Ministry of Health, 88590 Kota Kinabalu, Malaysia; 4 Faculty of Social Sciences and Humanities, Universiti Malaysia Sabah, 88400 Kota Kinabalu, Malaysia; 5 Johns Hopkins Center for Communication Programs, Baltimore, MD USA; 6 Department of Behavioral and Community Health Sciences, University of Pittsburgh School of Public Health, Pittsburgh, PA 15261 USA; 7 Mahidol Oxford Tropical Medicine Research Unit, Faculty of Tropical Medicine, Mahidol University, Bangkok, Thailand; 8 Centre for Tropical Medicine and Global Health, Nuffield Department of Medicine, University of Oxford, Oxford, UK; 9 Nord University, Bodø, Norway; 10 Department of Pathology and Microbiology, Faculty of Medicine and Health Sciences, Universiti Malaysia Sabah, 88400 Kota Kinabalu, Malaysia; 11 School of Biodiversity, One Health and Veterinary Medicine, University of Glasgow, Glasgow, UK; 12 Faculty of Infectious and Tropical Diseases, London School of Hygiene and Tropical Medicine, London, UK; 13 Saw Swee Hock School of Public Health, National University of Singapore, Singapore, Singapore; 14 Division of Molecular Parasitology, Proteo-Science Center, Ehime University, Toon, Ehime 791-0295 Japan; 15 The Ethox Centre, Nuffield Department of Population Health, University of Oxford, Oxford, UK; 16 Department of Public Health Medicine, Faculty of Medicine and Health Sciences, Universiti Malaysia Sabah, 88400 Kota Kinabalu, Malaysia

**Keywords:** Social inequities, Disparity, Malaria prevention, *P. knowlesi* malaria, Kudat, Sabah, Malaysia, Qualitative method, Photovoice

## Abstract

**Background:**

The increasing incidence of *Plasmodium knowlesi* malaria poses a significant challenge to efforts to eliminate malaria from Malaysia. Macaque reservoirs, outdoors-biting mosquitoes, human activities, and agricultural work are key factors associated with the transmission of this zoonotic pathogen. However, gaps in knowledge regarding reasons that drive malaria persistence in rural Kudat, Sabah, Northern Borneo remain. This study was conducted to address this knowledge gap, to better understand the complexities of these entangled problems, and to initiate discussion regarding new countermeasures to address them. This study aims to highlight rural community members’ perspectives regarding inequities to health relating to *P. knowlesi* malaria exposure.

**Methods:**

From January to October 2022, a study using qualitative methods was conducted in four rural villages in Kudat district of Sabah, Malaysia. A total of nine in-depth interviews were conducted with community and faith leaders, after the completion of twelve focus group discussions with 26 photovoice participants. The interviews were conducted using the Sabah Malay dialect, audio-recorded, transcribed, and translated into English. The research team led the discussion and analysis, which was approved by participants through member checking at the community level.

**Results:**

Participants identified disparity in health as a key issue affecting their health and livelihoods. Injustice in the social environment was also identified as a significant challenge, including the importance of listening to the voices of affected communities in disentangling the social and economic phenomena that can impact malaria control. Specific concerns included inadequate access to health-related resources and degradation of the environment. Participants recommended improving access to water and other necessities, increasing the availability of malaria control commodities in healthcare facilities, and developing sustainable programs to reduce socioeconomic disparities.

**Conclusion:**

Inequities to health emerged as a key concern for malaria control in rural Kudat, Sabah. A locally targeted malaria programme cantered on improving the social and economic disparities associated with health outcomes, could be a potential strategy for malaria prevention in such areas. Community-level perspectives gathered from this study can be used as a foundation for future discussions and dialogues among policymakers and community members for achieving greater transparency, improving social equity, and interoperability in addressing *P. knowlesi* malaria control.

**Supplementary Information:**

The online version contains supplementary material available at 10.1186/s12936-023-04750-9.

## Background

Over the past decade, *Plasmodium knowlesi* malaria cases in the World Health Organization (WHO) Southeast Asia and Western Pacific regions have raised global public health concerns [1]. Malaysia has reported the highest number of cases globally despite successfully eliminating human malaria in 2017 [2]. From January 2018 to December 2021, an estimated 13,727 cases of *P. knowlesi* malaria were reported in Malaysia, with the majority of cases detected in Sabah and Sarawak, both located in Malaysian Borneo Island [3]. The *P. knowlesi* malaria parasite is transmitted via the *Anopheles* mosquito of the *Leucosphyrus* group, when the female vector bites infected individuals of the natural reservoir, *Macaca fascicularis* and *Macaca nemestrina* monkeys [4], and subsequently bites a human [5]. This malaria vector is simio-anthropophilic, favouring monkeys and human blood [5]. While the majority of cases in humans have been mild, including symptoms such as fever, myalgia, abdominal pain, and vomiting [6, 7], fatalities have been reported in Sabah, with an overall case fatality rate of 2.4 over 1000 cases during 2010 to 2017 [6]. From 2019 to 2021, six and thirteen mortalities were reported in Sabah and Sarawak, respectively [3].

During the COVID-19 pandemic, Malaysia recorded 3575 *P. knowlesi* cases in 2021 [3]. The number did not differ significantly from previous years; the majority were detected in Sabah and Sarawak [3]. Historically, cases increased to around 2000 from 2011 to 2016 from a mere 356 cases in 2008 and have doubled to around 4000 from 2017 onwards [3]. High-risk groups include adults, the male gender, and people working in the agricultural sectors [8]. However, asymptomatic cases have been detected in all age groups and across genders [9, 10]. Fornace et al. reported a 6.9% prevalence of asymptomatic *P. knowlesi* cases in a population-based study done in Kudat and Kota Marudu, located in the Northern Borneo areas [9]. Communities can be at risk of contracting *P. knowlesi* malaria even if individuals do not venture into the forest [9]. Previous studies in neighbouring countries have highlighted the influence of socio-cultural and socioeconomic factors on malaria exposure. In rural communities in India, different perceptions of malaria and affordability issues in purchasing bed nets for larger households are challenging for malaria control [11]. Different beliefs concerning malaria transmission and poor access to health services in the Philippines challenge malaria control [12]. In Indonesia, workers who work in deep forests and along the forest fringe for weeks to months rarely use mosquito prevention products [13]. In Malaysia, the *Orang Asli* tribes in Peninsular Malaysia, are at risk of malaria transmission due to their lifestyle and housing structure, predisposing them to mosquito entrance. Their houses are built using wooden products, and the *Orang Asli* go into the forest for work activities [14]. These studies highlight the complexity of controlling zoonotic malaria, which requires an expansion of strategies beyond biomedical and environmental factors to include social determinants of health.

Globally, vulnerable communities living in rural areas, particularly those in or near forests, are disproportionately affected by *P. knowlesi* malaria [15]. Social disparities exist in communities living in rural areas and the forest fringe [16]. Vulnerable communities, such as the socio-economically disadvantaged *Orang Asli* in Peninsular Malaysia and rural communities in Sabah, Malaysia, are equally exposed to this zoonotic infection [17]. The high-risk population in Kudat, Sabah, lives in areas with a high proportion of absolute poverty, estimated at 41.5% of the total population, in which the household income could not meet the basic needs of food, shelter, and clothing [18]. Human malaria incidence is exceptionally high in the world’s poorest countries [19]. Regretfully, malaria control programmes often underserved the poorest and rural populations [20].

The dramatic rise of *P. knowlesi* malaria in recent years raises concerns about the effectiveness of current measures, such as insecticide-treated bed nets (ITNs), indoor residual spraying (IRS), and mosquito repellents or sprays [15]. Low socio-economic status has been associated with non-compliance with vector control measures [21]. Moreover, there is evidence of substandard healthcare in rural and vulnerable communities in several locations, especially in Malaysian Borneo, despite universal health coverage (UHC) in Malaysia [22]. These disparities in quality of healthcare services can lead to differential rates of mortality [6]. For example, issues have been raised about the unavailability of intravenous artesunate for severe malaria, delayed diagnosis, unavailability of specific and sensitive diagnostic tests, and barriers to healthcare access in Sabah [6]. Thus, it is crucial to explore the perspectives of the local community to better understand the reasons behind the ineffectiveness of malaria interventions. Qualitative research can explore the underlying reasons for the varied usage of malaria interventions [23, 24]. There is a need to fill this gap and explore the reasons behind zoonotic malaria exposure and its relation to human behaviour in vulnerable communities.

Much of the existing information on *P. knowlesi* malaria exposure in low socio-economic areas suggests risks related to incomplete housing structures and/or traveling to the forest or forest fringe for work. These ideas raise questions about inequities, defined as disparity or avoidable injustice that are unfair and unjust in a population in these communities arising from cultural exclusion, corruption or poor governance [25]. The term equity is often used as a synonym for inequality [26]. To relate to the study, the demarcation between these two terms can be differentiated; for example, inequality can be used when framing the differences between males versus females in getting malaria exposure. In comparison, equity is an ethical concept derived from the principle of social justice and human rights [26]. It focuses on the unequal distribution of resources or opportunities of individuals or groups [26].

Understanding the intersection between inequities and malaria exposure can yield answers to inform more holistic intervention strategies. Social, economic, political and public policy plays a significant role in shaping these factors [27]. The emphasis should be placed on allocating resources, such as bed nets or malaria awareness progress, identifying and overcoming barriers and facilitating the population’s needs [28]. This necessitates addressing inequity issues and moving beyond solely focusing on the disease. For instance, it involves directing resources towards disease prevention across multiple sectors, not limiting it to primary healthcare, and fostering local empowerment and participation in malaria program planning [27]. However, limited number of studies have specifically explored the social context and the persistence of *P. knowlesi* malaria in vulnerable communities. Therefore, to accelerate malaria elimination in Malaysia, the inclusion of community in research and the efforts to understand the local social challenges could be helpful. This approach could indirectly expedite-designing of locally targeted intervention and advance social equity and justice [29].

## Methods

### Study aim

This study was undertaken to directly raise a voice concerning malaria prevention from community members residing in rural villages in Sabah, Malaysia. The study was conducted in areas where previous *P. knowlesi* malaria studies have shown the degree of high-risk communities’ exposure to malaria. The study explores and disentangles the intersection between social inequities on malaria exposure, a topic that has only been explored to a minor degree in the scientific literature previously.

### Study design and eligible participants

The study utilized qualitative methods, including a photovoice study and in-depth interviews (IDIs), from January to October 2022. The photovoice study involved 26 participants from four rural villages in Kudat, Sabah [30] (Additional file 1). Subsequently, IDIs were conducted with eight community leaders and one faith leader, chosen through purposive and snowball sampling methods based on their roles as authorized representatives in the photovoice study villages. The IDIs expanded the information gained from the photovoice study from the perspective of the community leaders.

This recruitment strategy was guided by the information power approach, which aims to maximize the information obtained from the selected participants through five dimensions [31]. These include the specific aim of the study, the purposively selected participants, the adapted theory underpinning the study, the quality of communication and discussion between the researcher and participants, and the analysis process [31].

Before the interviews, the interviewer attended qualitative study and interview workshops and conducted a pilot study with local Sabahan colleagues to improve the IDI and FGD protocol and quality. Participants were recruited from four villages with a high incidence of *P. knowlesi* malaria in 2021 (unpublished record from Sabah state health government) as they could most address the research questions based on the local context. The information power approach suggests that the richer the data generated from the study, the lesser number of participants required [31] (Table 1).

**Table 1:** Eligibility criteria

The eligibility criteria for participants were:
FGDs with photovoice study participants (n = 26)
• Aged 18 years or older at the time of data collection in 2022
• A permanent resident of one of the four selected villages, defined as residing there for the previous 3 weeks
• No known comorbidity associated with cognitive function or psychological issue that could impact the study progress, such as documentation of photographs and participating in focus group sessions)
• Fully vaccinated for COVID-19
• Consented to participate in the study
• Those who were not permanently staying in the village due to travel to other districts for work or educational purposes were excluded
• Participants who were unable to attend and complete the photovoice discussion, whether online or physically, were also excluded
IDIs with community leaders (n = 9)
• Individuals^a^ who held positions as community leaders at the time of the study (e.g., head of the village, *Jawatankuasa Penduduk dan Keselamatan Kampung* (English: Village Safety and Development Committee), faith leader)
• Fully vaccinated for COVID-19
• Consented to participate

^a^Defined as an individual who can make decisions for the village/community members and/or a person who was elected/selected to hold an important position in the community

All IDIs and FGDs were conducted using a semi-structured interview protocol in Malay language (Additional file 2), and audio recordings were taken. The interviews were mainly conducted in Malay language and supplemented with Sabah Malay dialect at certain times. Field notes were taken to capture information related to the IDIs and FGDs in Malay.

### Study settings

**Fig. 1:**
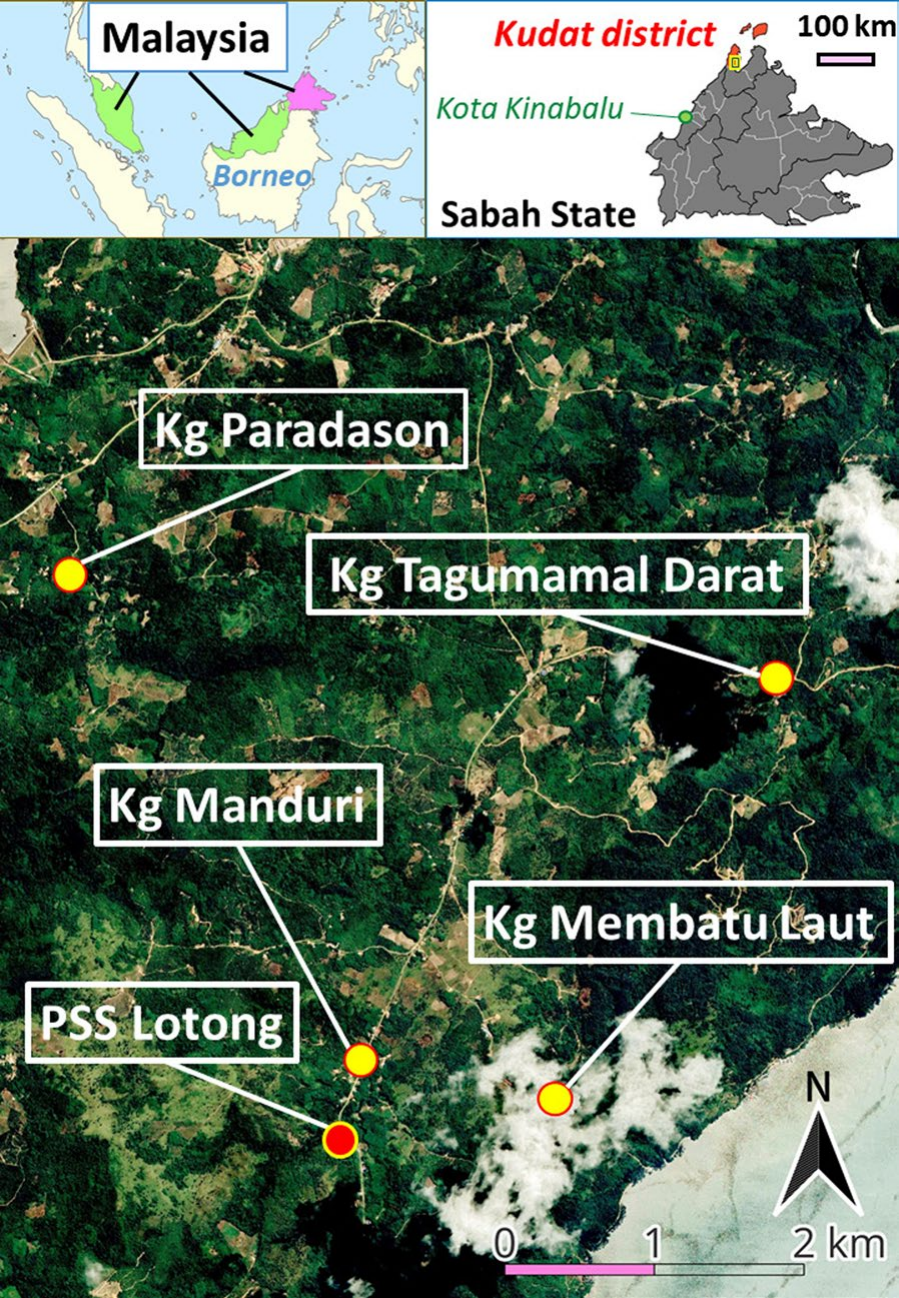
The study sites

The study location is Kudat, which is a district located in the North-division of Sabah, Malaysia. Kudat has an estimated population of 86,410 inhabitants [32] (Fig. 1). The study area has environment that is suitable for disease transmission due to the presence of *Anopheles* mosquito from the *Leucosphyrus* group, and macaque monkeys are migrating nearby human settlements, likely as a result of anthropogenic activities, such as deforestation [33]. Before conducting this study, the research team proactively engaged with the gatekeepers to encourage effective participation before conducting this study [16].

Household Income and Basic Amenities Surveys (HIS) show that Kudat has an estimated 41.5% of the population living in absolute poverty [18]. Much of the population work as farmers and rubber tappers [34–37]. In general, Kudat and its neighbouring districts, Pitas and Kota Marudu, have a high proportion of communities with a low socio-economic level [8]. In Kudat in 2019, the estimated median annual household gross income was RM2592 (estimated USD613.52) compared to RM2480 (estimated USD586.85) in 2016 [18]. Compared to Peninsular Malaysia, socio-economic inequities are relatively common throughout Sabah [38]. In Kudat, the vast majority of households have a water supply to their home (85.6%) and access to electricity (99.8%) [18]. Access to healthcare centres is limited due to the low percentages of households with a car (52.5%) and motorcycles (20.4%) [18]. Only 20.2% of households in Kudat have garbage collection facilities [18], which might contribute to mosquito breeding sites in areas where garbage is thrown as immature forms of *Anopheles balabacensis* occupy shaded and temporary freshwater pools [39].

### Data collection

The data collection for this study was conducted during Malaysia’s National Recovery Plan (NRP) in response to the COVID-19 pandemic. Standard COVID-19 prevention measures were followed to ensure the safety of both the research team and the participants. These procedures included wearing face masks, conducting research in open areas or rooms with good air ventilation, and ensuring that the research team and participants were fully vaccinated. The session was postponed if any participant or research team members showed symptoms such as flu or cough.

Due to poor internet connection in the study areas, all interviews were conducted face-to-face at the study sites, in places that were comfortable for the participants. Both IDIs and FGDs were conducted, with each session lasting approximately 60 to 90 min. The interview protocols were approved by a supervisory committee at Universiti Kebangsaan Malaysia (UKM), while the research was approved by the research approval boards at Universiti Kebangsaan Malaysia and the Ministry of Health Malaysia.

All study data were saved in a password-protected file that was only accessible by the research team. To ensure the confidentiality of the participants, all identifiers were changed into pseudonyms.

### Data analysis

The themes were generated using reflexive thematic analysis [40]. Initially, N.A.N. reviewed and familiarized herself with the dataset by transcribing the data into interview transcripts. Re-reading the transcripts allowed explicit and implicit codes to be generated from the data. The themes were reviewed iteratively during the discussion with participants as a form of member checking. M.R.H, R.H, and P.Y.P.L then reviewed the themes to increase the richness of the data. N.A.N., M.R.H, R.Z., P.Y.P.L, defined the themes, and all co-authors collaborated in the writing of the article. All the recordings were transcribed word by word, and only relevant quotes were translated into English. The Sabah-Malay-dialect to English translations were verified by P.Y.P.L, to ensure the translations were accurate and retained the nuanced meaning from the original responses.

Throughout the study, rigor was assured using several approaches [41]. Firstly, the research team built relationships and trust with the community leaders in the study area prior to the study [16]. Secondly, photovoice and IDIs participants were purposively and specifically selected, as they could address the local issues related to the research inquiries from the perspectives of residents living in the village. The study was credible as triangulation was done through the use of various methods used to generate the study data and through different participant perspectives. Thirdly, N.A.N. conducted the analysis with support from co-authors who were experts in their field (M.R.H., R.H., P.Y.P.L) and received support from all co-authors who were experts in their field [41]. Fourth, to achieve conformability, the study involved the use of the direct words of the participants through integration of participants’ quotes. Finally, with the detailed descriptions generated throughout the study, transferability could be established for future research to adapt and replicate [42].

## Results

The first major theme noted from the research data was social disparities in health in rural communities. Within this theme are five subthemes: (i) water access challenges increase malaria risk in rural areas; (ii) impact of limited access to electricity on malaria prevention in rural areas; (iii) unequal internet access in rural areas poses health risks; (iv) limited access to healthcare and resources in rural areas hinders malaria control; and (v) the neglect of community concerns, lack of policies and capacities that hinder malaria control. The next major theme is the livelihood challenges to malaria prevention due to local socio-economic structures and practices followed by environmental degradation and malaria risk in rural communities (Additional file 3).

### Theme 1: Social disparities to health in rural communities

#### Subtheme: Water access challenges increase malaria risk in rural areas

Across communities, participants revealed that even though water pipes are present, treated water is not continuously supplied, resulting in limited access to water throughout the villages. Unfortunately, this issue could considerably increase the likelihood of malaria transmission due to mosquito exposure. A faith leader explained,

“*People whose houses have piped water also have persistent water supply issues. Villagers have to seek a water source at the streams and rivers. There are many mosquitoes there. How do we avoid malaria?*’’ [Faith leader, IDI05].

Furthermore, the participants from Kampung (Kg) Membatu Laut explained that since the lack of water has been ongoing for a long time, the communities have put effort into creating a water supply through a water gravity system, which has its main source point located in the forest. The gravity water requires a strong water current to supply to the houses. However, the system has limitations, as the supply issue remains when a house is far from the main water point. During the in-depth interview, a pastor noted however,

“*Even though there is a gravity water system, when the water source is not strong, and many people are using the water, there are times when the supply is not enough for everyone*.’’ [IDI05].

**Fig. 2:**
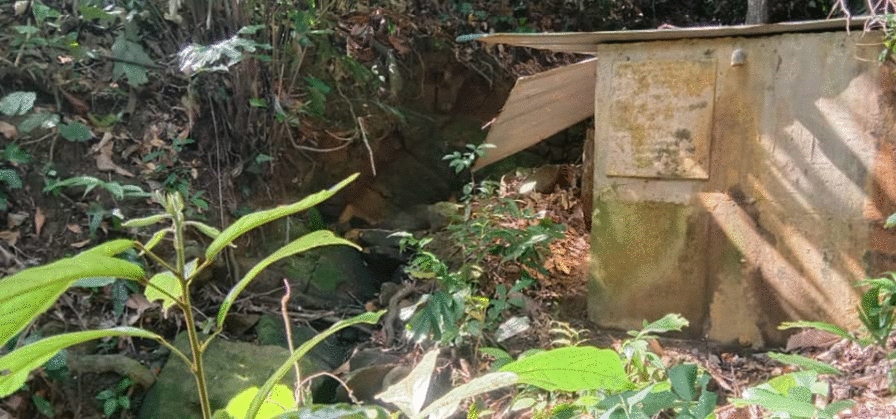
“The “lokos”. We need to go there. Our main water source” [Female, Farmer, Kg Paradason]

To address the lack of water pipes, participants from Kg Paradason revealed important insights and discussed “*lokos*”, a local term describing the water source in the forest, similar to a spring. Villagers are required to walk for almost 20 to 30 min to get to the area. They also pointed out that the area around the “*lokos*” and surrounding small ponds had many mosquitoes and larvae, making access to these areas a health risk (Fig. 2).

#### Subtheme 2: Impact of limited access to electricity on malaria prevention in rural areas

**Fig. 3:**
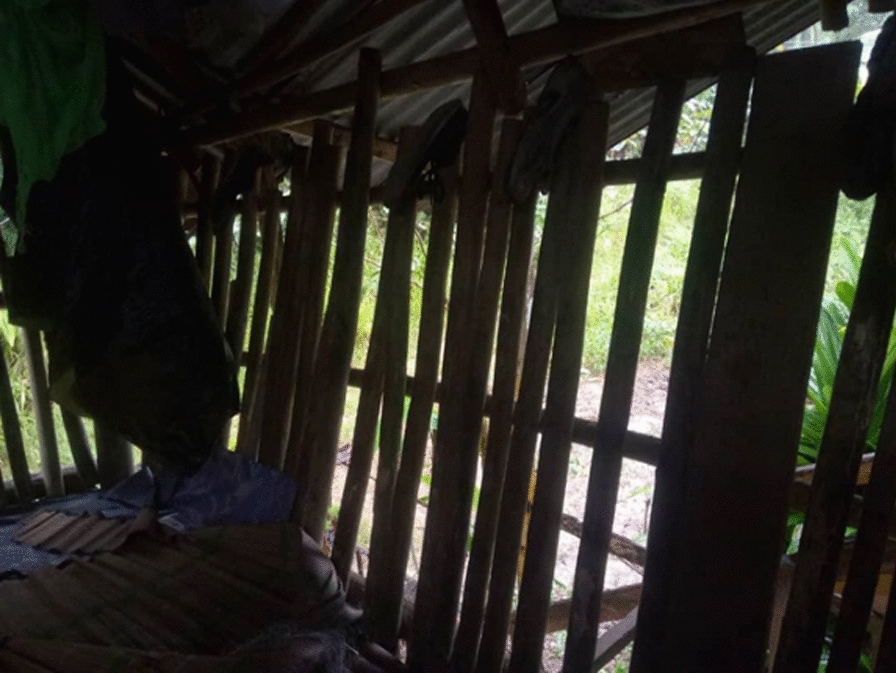
“It always feels like night time here” [Male, Farmer, Kg Paradason]

Participants from Kg Paradason drew attention to the critical issue of electricity supply in their village, which related to being able to control mosquitoes. Participants shared their experiences of constantly being surrounded by mosquitoes inside and outside their homes due to the dense forest covers that block out sunlight even during the day. This dark environment provides ideal conditions for *An. balabacensis* mosquitoes to fly indoors and bite humans, and return outside to rest [39]. A female participant, who had lived in the area for over 30 years, expressed her concern:

“*Our houses would be illuminated if we had access to electricity. However, unfortunately, we do not have electricity, and as a result, mosquitoes find their way inside our homes. It always feels like we are living in perpetual darkness here*’’ [FGD P2] (Fig. 3).

#### Subtheme 3: Unequal internet access in rural areas poses health risks

Participants across all study sites described the inconsistency of communication signals in their villages due to the inaccessibility of internet lines, leading them to travel into areas where mosquitoes are present, which in turn put them at higher risk of malaria. This limitation leads villagers to search for “line hotspots” in various locations, such as near the forest, in the plantation area, or on the hills, for instance, in Kg Membatu Laut, villagers have sometimes been able to get an internet signal from Pitas, a district located on the right side of Kudat and separated by Marudu Bay (see map in Fig. 1).

They added that schools and workplaces were closed during the pandemic when movement restrictions policies were implemented in Malaysia. People, including schoolchildren, were at risk of malaria while searching for internet lines to attend classes. Adults were also affected while searching for access to be online for personal reasons. Despite the presence of line substations in or near the village, the signals were weak or sometimes unavailable, forcing them to travel into areas at higher risk of malaria. A village administrative officer described it this way,

“*Some houses in certain areas around the village have no internet lines, even though there are substations nearby on the left or right side of the house*’’ [IDI09].

#### Subtheme 4: Limited access to healthcare and resources in rural areas hinders malaria control

The participants noted that a major concern was the absence of a hospital in their villages. The nearest district hospital, without a specialist, is located around 30 to 40 min away by car (estimated 15 km). Primary healthcare services and malaria control were provided by a clinic located in Kg Lotong. However, participants expressed their worries that some people in the village might have difficulty seeking malaria diagnosis and treatment due to the distance to the healthcare facilities from home, time limitations, and unavailability of transport or assistance to send them to the clinic. A pastor from Kg Paradason stated,

“*Do you know it is difficult for the villagers to visit the clinic? Some people do not have transport. The clinic is far. When it rains—it makes it more difficult. People could not walk there. The people must think about who will take care of the children if he or she goes to the hospital. So people will stay home and not go to the clinic*.” [IDI05].

Additionally, participants described their challenges in obtaining new bed nets and explained that limited resources at the clinic could delay the distribution of bed nets to villagers. One female participant from the village shared her experience.

“*I just returned from the clinic this morning to get the bed net. They (the healthcare worker) informed me they wanted to distribute to us, but they have not come, so I went there and took it myself*” [Female, Kg Paradason] [FGD P1].

Moreover, despite the provision of bed nets, participants revealed that it was not easy to replace a bed net if it was torn. The affordability of purchasing extra bed nets and other anti-malaria items was viewed as an issue for many participants. Despite these limitations, the healthcare team continuously puts effort into providing the best healthcare to the villagers, even during the COVID-19 pandemic. Malaria officers were described as providing support through screening the villagers, conducting “house” spraying, and updating villagers with information if there were any malaria cases in the village.

Furthermore, participants explained that some villagers still seek care from traditional healers for their illnesses as an alternative to the formal health sector. This health-seeking practice offered support to some villagers despite the need to travel for the traditional healer living near the formal health clinic or neighbouring village.

#### Subtheme 5: The neglect of community concerns, lack of policies and capacities that hinder malaria control

Participants identified the lack of supportive policies, conflicting agendas, and limited government and institutional capacities to support their needs as barriers that hamper effective malaria prevention. Some participants said that various stakeholders would often ignore their concerns about the community’s exposure to malaria. Community leaders emphasized the importance of sharing research findings and the latest knowledge on malaria with their communities. Due to their ongoing concern, community leaders frequently raised concerns over water scarcity issues and monkeys near human settlements during district meetings.

**Fig. 4:**
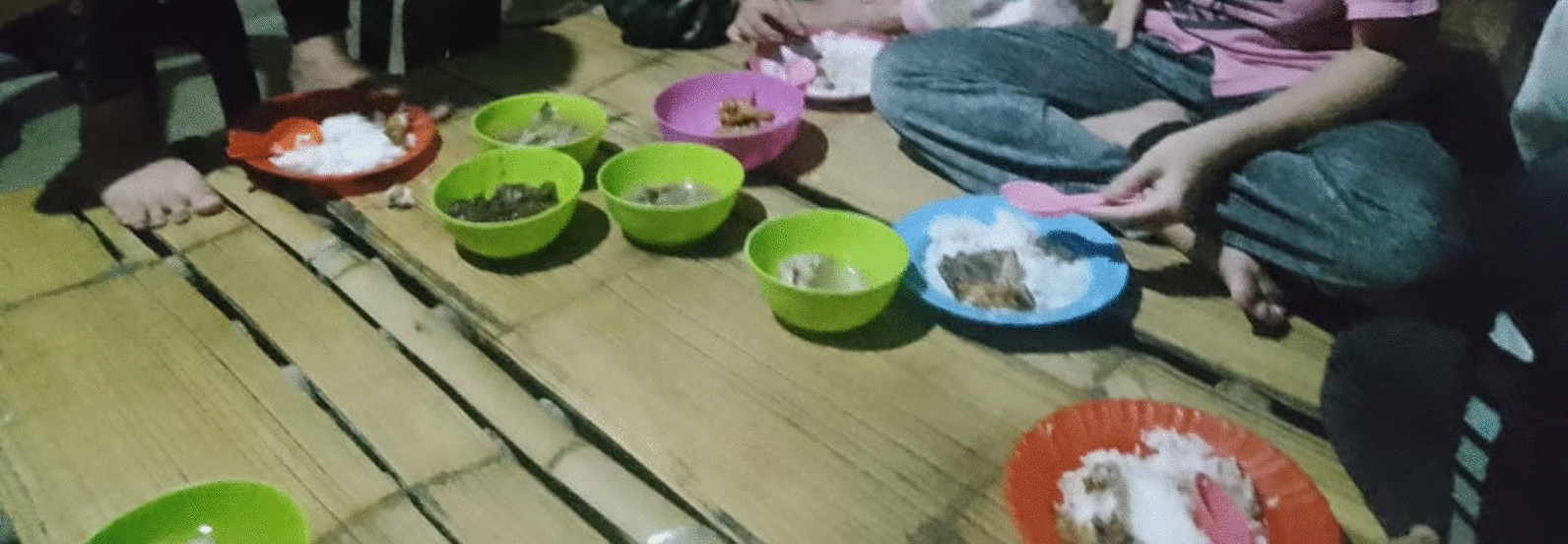
“A simple dinner for my family at our home” [Female, Farmer, Kg Paradason]

Participants explained the critical need for better housing conditions to help reduce the risk of mosquito bites, as many villagers’ houses were built using bamboo, rattan, and forest materials. The structure of the walls, windows, and ceilings of their homes expose them to mosquito bites. Participants highlighted the importance of the government’s *Projek Perumahan Rakyat Termiskin* (PPRT) (Home Initiative for Extreme Poor). They described the PPRT as a unique government program to support the need of poor villagers to build a house for their families. Participants shared that the PPRT house could provide comfort to their family and prevent malaria by having brick walls with proper doors and windows. Electricity, water supply, and a bathroom were known to be provided. One female participant from Kg Paradason shared her hope that the government will approve their application for this program to improve the living conditions of their families and future generations, as shown in Fig. 4.

Participants also described their experiences of explaining their opinion to previous researchers who had visited their village. They highlighted how monkeys disturbed their crops, farms, and fruit trees, even entering their houses and kitchens. They used the term ‘*kera*’ or ‘*monyet*’ when describing such encounters with the long-tailed monkeys. The ‘*monyet’* was also described as eating chicken eggs and fruits from the oil palm trees. A community leader from Kg Membatu Laut expressed his frustration, stating,

“*I asked them (the researchers and officers from many organizations), can we shoot the monkeys? We wanted to kill them because these animals (monkeys) disturbed our farms. They replied, ‘You cannot kill them […], so when they answered as such, what can we do? So, you see, no one is taking care of this issue. Do we leave the issue here? Let the monkey disturb our farm. Can anyone help us and take some action? The paddy (padi) and corn took a year to grow and were supposed to be the food source for a year. However, they were destroyed by the monkeys.*’’ [Community leader, Kg Membatu Laut] [IDI04].

### Theme 2: The livelihood challenges to malaria prevention due to local socio-economic structures and practices

**Fig. 5:**
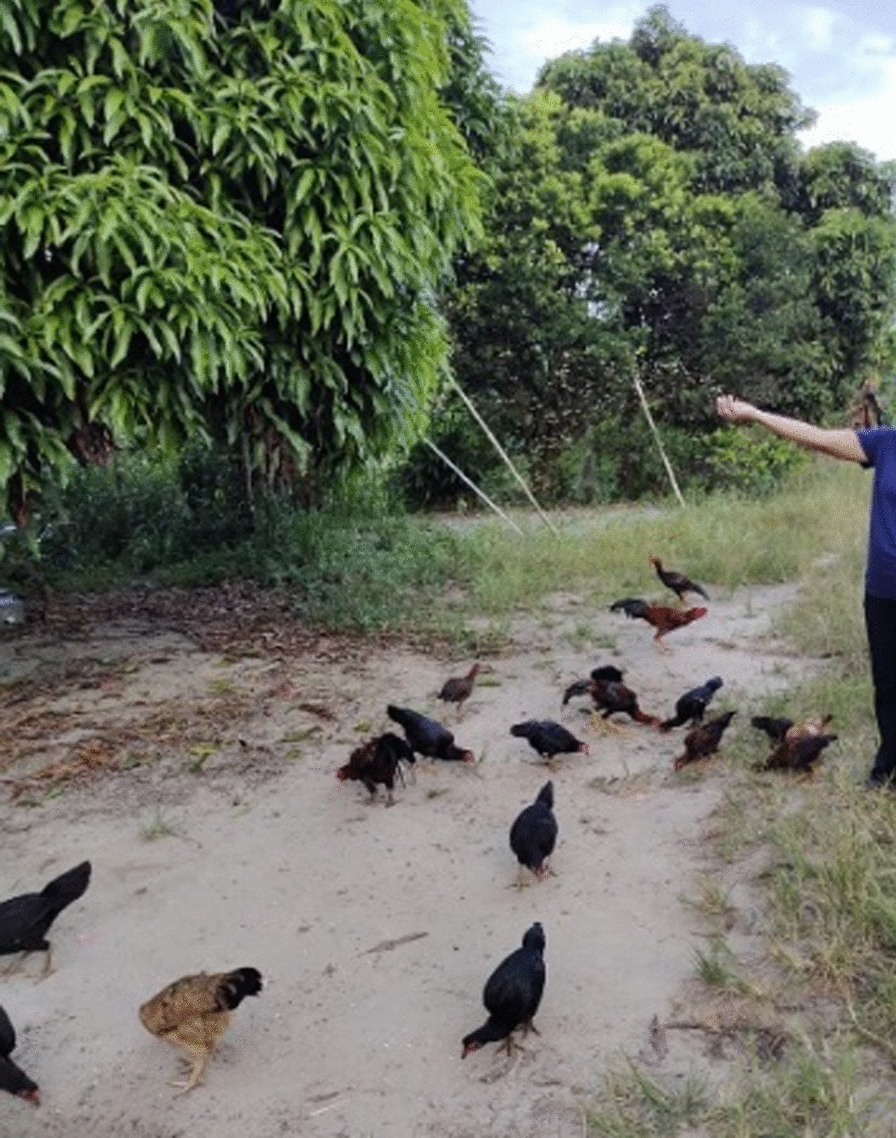
“Feeding chickens is a routine for some of the villagers here” [Female, Self-employed, Kg Membatu Laut]

The second important theme generated from the research data was the challenges to malaria prevention due to local socio-economic structures and practices. The communities are commonly exposed to malaria based on the sections they work in. For example, most people worked in the agricultural sectors such as rubber tapping, picking coconuts, working in oil palm plantations, and farming. Other livelihood activities were fishing, finding non-timber products in the forest, and breeding chickens. These jobs expose community members to malaria, as shown in Fig. 5. A community leader from Kg Paradason explained,

“*It is not easy to avoid malaria in this village. People need to go out, to search for vegetables, food, and their living. People do activities around the village. People who have chicken, they will feed them in the morning and the evening*’’ [IDI02].

**Fig. 6:**
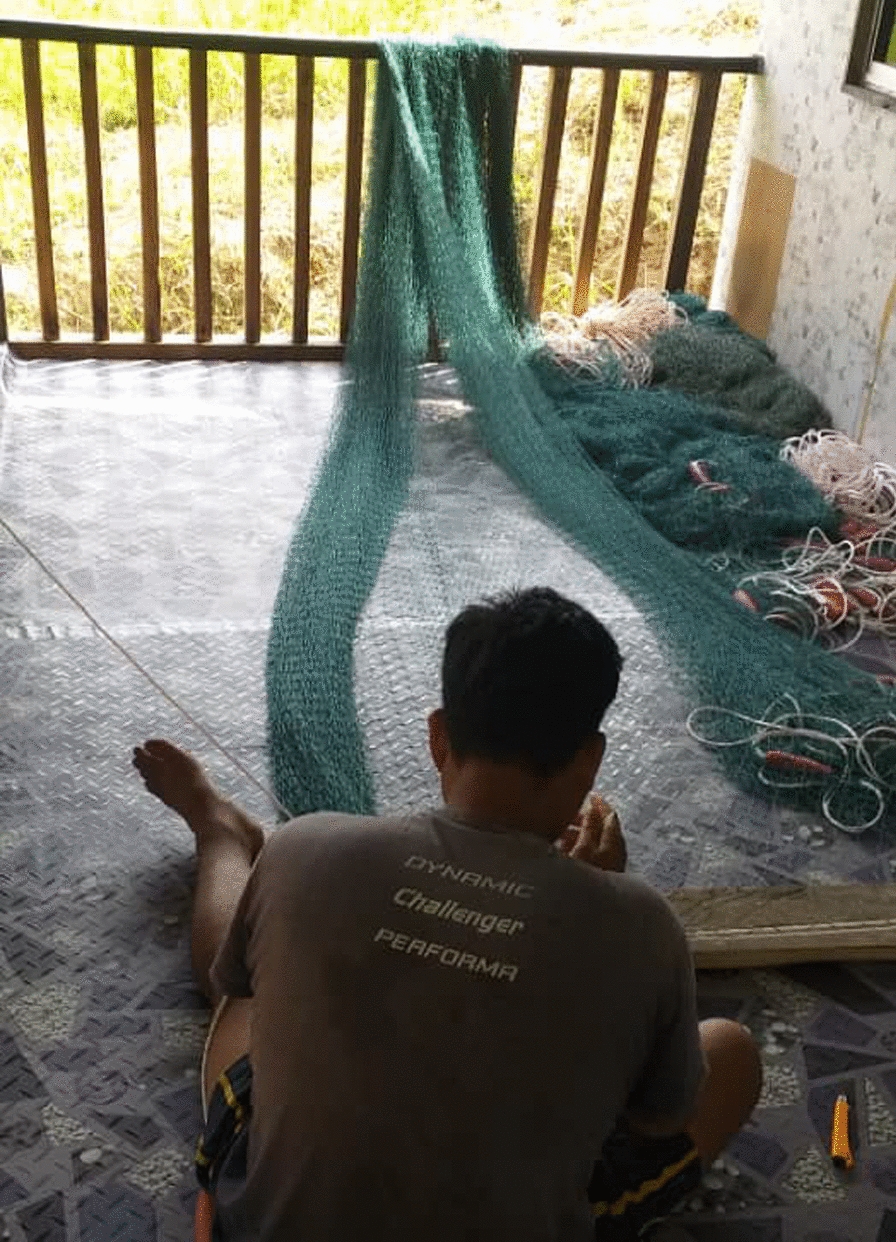
“One activity the people in our village do is tending to their fishing nets from evening to night time after they returned from the sea” [Female, Farmer, Kg Membatu Laut]

In general, most current vector controls are impractical for their working situations. The current bed net design could be re-designed into a more practical product, as currently, the bed nets are not suitable for outdoor activities or work (see Fig. 6).

“*The bed net could be re-designed, making them into clothing that can be worn during work. The current bed net design was not feasible during outdoor activities […] How can someone wear the bed net in the forest and while working? The dressing would only make it get stuck to the trees and branches in the forest?*’’ [Village administrative officer, Kg Paradason] [IDI08].

Participants highlighted issues with the smells produced by mosquito control items that conflicted with their work. For example, some participants explained the difficulty of using repellents during activities like hunting. Participants explained that hunters often would not put on mosquito repellents as the products produced a smell that made the animals avoid the area as they could sense the smell from afar. Likewise, the hunters would not bring bed nets, as they need to be mobile.

Villagers who work on farms may spend their nights in small huts called “*sulap*”, which puts them at risk of malaria. As explained by one female participant from Kg Paradason,

“*My husband, I think he was infected with malaria because he went to the forest to get the materials to build the “sulap”* (English: hut).’’

**Fig. 7:**
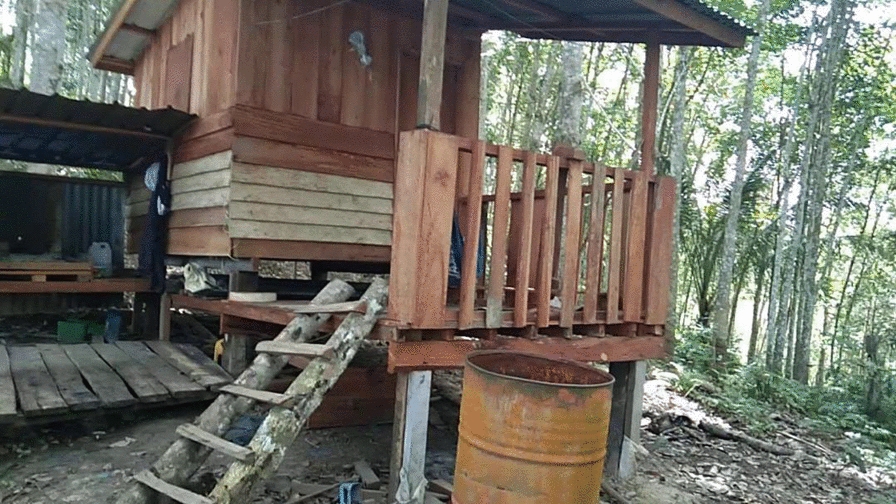
“A “sulap” in a farm” (Female, Housewife, Kg Paradason)

When the villagers would go to protect their fruit trees and crops from wild animals, including monkeys, they exposed themselves to malaria (see Fig. 7).

The participants’ livelihoods depended on their daily income. Despite knowing the malaria risk, participants highlighted that working and earning a living is more important for the villagers. They use protective clothing, long trousers, and repellents to minimize the risk of mosquito bites. However, some still get infected with malaria.

“*Everyone knows about malaria. Villagers who go for rubber tapping go to their farm early in the morning, around 3 or 6 o’clock. Some even go during the midnight hours. Time is crucial because it affects the production of latex. Everyone knows that we need to avoid mosquitoes. However, what can we do? I always think about how we can avoid this contact with mosquitoes. These people, the villagers, still need to go out, work, and live. We open the farm, do rubber tapping, and search for vegetables. How could people prevent it? Of course, wearing protective clothing, long pants, covering the head, putting on the repellents, but some people still got infected by malaria.*’’ [Community leader, Kg Membatu Laut] [IDI04].

Many participants described the difficulty of measuring household income because they needed to work multiple sources of income to support their families. One participant said her daily routine involved going to the farm in the morning before seven o’clock, sending her kids to school, and returning to the farm afterward. These activities could be influenced by season and year as some individuals will go to a different district to work. Thus, household income was difficult to measure; moreover, it differs according to gender.

### Theme 3: Environmental degradation and malaria risks in rural communities

The communities in the study sites have a close relationship with the natural environment, where forests, plantations, and farms are integral parts of their everyday lives. However, community leaders have noted that the natural forests have been diminishing over the years due to the conversion of land into plantation and farming areas. This loss of habitat may have contributed to the presence of monkeys in and around their villages, which in turn has increased the risk of “monkey malaria.” The risk is further amplified due to the lack of proper roads in the villages, which creates temporary water pools suitable for mosquitoes to breed. To minimize the risk of “monkey malaria,” one community leader advised,

“*To avoid monkey malaria, do not plant fruit trees or oil palms near the village. Monkeys nowadays eat oil palm fruit. If we talk about the monkeys, this is a very complex and challenging issue. It is not easy to keep them away from us. There are many of them, and as time passes, their quantity can get higher than today*.’’ [Community leader, Kg Tagumamal Darat] [IDI03].

Similarly, another community leader noted,

“*There are many water ponds in our village, especially from the tire track, where mosquitoes could breed*” [Community leader, Kg Paradason] [IDI02].

Another community leader expressed concerns about the impact of projects around their village, as there have been no detailed discussions with the community. They worry about the future generations of the village and urge those with decision-making power to listen to their voices and address the challenges faced by the villagers,

“*I do not want to focus on those lands that have undergone deforestation directly*, but *I hope those with the power will listen to our voices. Listen to the challenges faced by the villagers. It is not easy for us to feed our family, dealing with health issues and the challenges of living life nowadays. This is critical to discuss, not for our benefit, but for future generations. This is not just for today but for years to come. People need to think what will happen here, in ten years in the future*” [Female, Village administrative officer, Kg Manduri] [IDI01].

### Recommendations by the study participants

#### Improving the infrastructure and access to necessities, promoting good health, and government support

Participants expressed their aspirations for a better social environment, including access to treated water and internet/communication lines. However, one participant mentioned that the road’s poor condition hindered the sewage system and water supply development. He stated:

*“There is no proper road for the tractors to come here. There is no road here. But small, village road. I frequently sent my application to those in charge to send their support to these needs. However, they answered, ‘You have to wait’. Wait. Wait. So, I waited. And I waited until now, but nothing was done here in our village*” [Male, Village administrative officer, Kg Paradason] [IDI08].

They requested the government to continue the piped water project that had been stopped for a long time and to support building brick houses, which could reduce the risk of mosquito bites:

*“The government could continue the project that was stopped. Please continue with the piped water project that has been stopped for a long time. When they complete the project, villagers will get a good water source. The government also can support building brick houses that could reduce the risk of mosquito bites. For example, a proper toilet in the house reduces the risk of malaria as people do not have to go out for the toilet. This way could help to ensure people would not go out to seek for a water source for many other reasons.*’’ [Pastor, Paradason] [IDI02].

Participants also highlighted the significance of the government and other sectors in managing garbage disposal and environmental cleanliness issues. They emphasized the importance of reducing mosquito breeding sites by regularly cleaning areas such as the roadside and river:

“*I would like to highlight the importance of reducing the mosquito breeding sites in and around our village. These areas require regular cleaning. This includes the roadside and the river. It could help with the stagnant water issue and reduce the mosquito breeding sites.*” [Male, Village administrative officer, Kg Paradason] [IDI08].

#### Addressing disparities, reimagining innovative approaches, and collaborating for effective solutions

The participants hold varying opinions on innovative approaches to malaria control, influenced by factors such as socio-economic status, lifestyles, or socio-cultural activities. For example, one participant suggested cream and lotions:

“*I suggest the government provide free items such as creams or lotions to avoid mosquitoes to the villagers. The bed net is not helpful. People go to the farm, and they get bitten by mosquitoes. These people have already tried to avoid mosquitoes by using repellents or any do-it-yourself- measures. But, when the wind blows there, the smoke follows. If the cream is provided for us, I suggest we use them. We can apply it on the neck, on our hands*” [Male, Farmer, Kg Tagumamal Darat] [FGD TD3].

While participants acknowledged the importance of taking preventive measures against malaria, they also recognized that significant disparities exist. A community leader from Kg Membatu Laut emphasized medical intervention,

“*I believe the most effective way could be by vaccination. Why can they design the COVID-19 vaccine rapidly but not for this disease (malaria)?*’’ [IDI04].

Participants emphasized the importance of government and stakeholders’ support in addressing malaria-related issues. Although they appreciated the government’s efforts, they highlighted the need for more equitable social and economic policies to reduce the risk of malaria in their village. Participants believed that a collaborative approach involving government, stakeholders, and community members could be an effective strategy for improving malaria control in the villages and facilitating communication of the needs of everyone.

#### Community empowerment and cross-sectoral collaboration for effective malaria prevention

Participants emphasized the need for their voices to be heard and called for greater cross-sectoral collaboration on malaria prevention. While they appreciated the current malaria program’s efforts to assist disease prevention in their villages, they suggested that more could be done. Participants felt that the current relationship between community members and healthcare workers could be expanded to improve malaria control in the villages. For example, some participants expressed excitement at the prospect of being part of a research project that used photo exhibitions as a creative platform to raise their voices and concerns to a broader audience.

Participants highlighted the Ministry of Health and government sectors as the primary contacts to encourage and facilitate collaborations with different sectors. Participants also felt that their voices were often unheard due to their social status. For example, participants understood the need for malaria control by focusing on mosquitoes, but they also highlighted the need to find a solution to the monkey presence around their village. The monkeys were disturbing and destroying their fruit trees and crops, making them a significant obstacle to malaria prevention efforts. Participants made specific recommendations for improving malaria control in their village. For example, one community leader mentioned the quality of mosquito control products and the issues with monkeys:

“*The government needs to find ways to check on the quality of the given bed net, whether there is a need to change more frequently or design more likable bed net. Likable, I mean, not heaty, has airflow, and is easy to put on before sleep. Secondly, the house spraying. Maybe they need to check on that. Is it effective? I can still see mosquitoes flying even after the spraying. Thirdly, the monkeys. Someone needs to get rid of them. Or move them elsewhere. The villagers know about malaria […], but we are dependent on the government, or other sectors, anyone that could help us – to kill the “kuman malaria” [English: malaria agent] and to control the disease*” [Community leader, Kg Tagumamal Darat] [IDI03].

Another participant pleaded for support to address the monkeys and stagnant water as related to the risk of malaria by explaining:

“*If we want to make an effort to control this infection in our village, please do something about the monkey presence. Catch the monkey, I understand that it is impossible in a way, but please do something about it. These monkeys are the “perumah” (English: reservoir), and they come near to us humans […] This issue is very complex. The mosquitoes are here too, and they keep breeding around our village. Here, they have all the areas to breed, ponds, plantations, and farms. What I want to highlight here is that stagnant water is everywhere. Someone has to do something*” [Pastor, Kg Paradason] [IDI05].

Participants felt that collaboration between community members and stakeholders is crucial to develop effective malaria control solutions. They also emphasized the need to address the issue of the monkey presence in their village, in addition to traditional malaria control methods. Overall, their recommendations are critical for developing targeted interventions that address local realities and socio-economic factors.

## Discussion

This study identified and untangled a broad range of inequities issues to health relating to malaria exposure in rural communities exposed to *P. knowlesi* malaria in Kudat, Sabah. The study disentangled the underlying reasons behind malaria persistence and emphasizes the importance of exploring not only proximate causes but also root causes of malaria persistence. Inequities issues were critical factors in *P. knowlesi* malaria exposure and should be considered in malaria interventions. Limited resources and disparities in rural communities, livelihood challenges due to local socio-economic contexts, and environmental degradation, which negatively impact the environment and health, are some of the inequities issues in rural communities exposed to *P. knowlesi* malaria in Kudat, Sabah. This study provides an emerging context, helps to fill gaps in understanding the underlying reasons behind malaria persistence in the study area, and emphasizes the importance of social justice issues in these communities.

There is a need to modify policies and tailor them to the need of rural communities and their microenvironment [42, 43]. Based on the concept of differential vulnerability by Diderichsen et al. [44], this study argued that intervention must look at different vulnerability factors and not only at single exposure. In disadvantaged populations, vulnerability can only be reduced when the social condition improves significantly or interacting exposures are diminished. Previous evidence suggests that improving social inequity issues can reverse malaria by improving socio-economic conditions [44]. Investing in livelihood improvement through local socio-economic development is equally important as compliance with existing malaria control measures to strengthen malaria intervention [45]. The relationship between malaria and low socio-economic status has often been described as a vicious cycle, with malaria resulting from low socio-economic status [46]. Improving the housing structure was protective against malaria and is an important example of long-term improvements that can reduce the risk of malaria [47]. Research suggests the housing structure should be well-built, with closed eaves, built using bricks, and have tiles, metal roofs, or ceilings [47, 48]. The PPRT, as shared by the participants in Kg Paradason could be a complementary tool in designing a paradigm shift for malaria control in the study area. There is a need to modify policies by tailoring them to the need for housing intervention based on local micro-climate to protect vulnerable communities against malaria [50]. Efforts should include improving the physical and social environment for communities exposed to *P. knowlesi* malaria. In sub-Saharan Africa, where communities are highly exposed to human malaria parasites, for example, *Plasmodium falciparum* and *Plasmodium vivax*, public policies were recommended to focus on housing conditions and improve income, wealth, and occupation for socially disadvantaged groups [51]. This form of investment could prove highly effective and sustainable against malaria in the long term [44, 47]. Such integration could support reducing the burden of malaria [45], and the overall effort to make settlements safe, resilient, sustainable, and inclusive in achieving the United Nation’s Sustainable Developmental Goals (SDG) 11 [52].

The study also uncovers the impacts of day-to-day life inequities that directly impact population health. The issue with access to fundamental needs to necessities like water and electricity is a chronic issue that is often overlooked. Unique to this study and highlighted by the COVID-19 pandemic was evidence of the lack of internet access as a contributing factor for malaria exposure, particularly for schoolchildren seeking internet ‘line’ (wi-fi signals) for school access. Actions in multiple sectors are needed to assist communities in meetings these fundamental needs [53]. While there may be concerns that providing electricity could attract malaria vectors and increase malaria risk [54], it is essential to carefully evaluate people’s circumstances and promote adherence to recommended malaria prevention behaviors [55]. The study also highlights the need for alternative approaches in zoonotic malaria control, including collaboration with non-governmental organizations to provide better access to clean water, electricity, and other fundamental basics for a living. Collaboration between researchers, policymakers, regulators, and communities is critical for developing innovative malaria interventions and sustaining malaria control and elimination efforts [56]. Previous evidence demonstrates that socio-economic intervention, such as improving housing structure, occupation, income, and wealth, can significantly reduce the malaria burden in sub-Saharan Africa [51], Sri Lanka, and other areas [56].

The recommendations highlight the need to engage with the community, government, and other stakeholders to support and innovate malaria control [57]. Examples of such conditions exist elsewhere; in India, the collaboration between academia and community members facilitated the initiation of the Vector Control Research Centre (VCRC), which integrated the plantation of algae to reduce the development of mosquito larvae and improve the socio-economic status of the villagers [58]. In return, these algae were used to make cardboard, and in sustaining this intervention, financial incentives were given for them to grow, harvest, and sell the algae [58]. In Malaysia, volunteer community health workers (CHWs), called the *Sukarelawan Penjagaan Kesihatan Asas* (*SPKA*), have continuously supported the need for early detection and screening of probable malaria cases in rural villages [59]. However, their job scope is limited. Policymakers and researchers should consider the role of community members and/or community healthcare workers as co-researchers by interviewing, conducting observations, and participating in research to explore the barriers to malaria prevention [60]. The need for a supportive enabling environment is essential by building local community capacity and sharing the programme ownership towards a more locally targeted malaria intervention and human-centred design [61].

Finally, by addressing the intersection between social inequities issues and vulnerability to malaria exposure, this study entangled an emerging context that could transform the understanding of the importance of social justice issues in these communities [62]. There is a requirement for social innovation [29, 63] and reinforcement of political will, better health guidance, strategic information dissemination and coordinated responses [56]. In line with the SDG’s goal to eliminate malaria by 2030, addressing malaria interventions should extend beyond a narrow focus on universal healthcare services. This expansion necessitates capacity building within healthcare services and the implementation of cross-cutting actions encompassing the diverse social, economic, political, environmental, cultural, and other determinants of health. Novel strategies must be developed to control *P. knowlesi* malaria effectively, prioritizing transparency, equity enhancement, and the integration of realities on the ground.

## Limitation and strength

Although the study was not designed to produce findings that are generalizable to other locations and populations due to its qualitative nature, it was able to include a range of perspectives by community leaders and members. The qualitative methods used generated insights into emerging perspectives on social inequities in rural communities exposed to *P. knowlesi* malaria, which had not previously been described. By integrating the perspectives of community leaders and members, the study provides a better understanding of the issue, which are transferable to other similar communities worldwide.

The study was limited by the inability of the research team to conduct additional interviews and observations in the field due to time limitations, geographical distance, and other resource restrictions. However, the study offers rich, visual representations of the current situation on the ground using a community-centred approach. Collaborating with participants, including ongoing connection and coalition building are a hallmark of socially just, environmentally, and economically just research [26, 55]. To continue building the relationship and producing outcomes from the study, a Facebook page called “KLIKMALARIA” has been created and is moderated by the communities.

## Conclusion

The study has revealed emerging and critical issues of inequities to health and malaria exposure among rural communities exposed to *P. knowlesi* malaria in Sabah, Malaysia. Given the significant impact of social and economic disparities on daily lives and livelihoods, innovative and sustained strategies are needed to address this issue. These strategies should involve the participation of community members, stakeholders, and policymakers and should focus on improving the access to health-related resources, as well as addressing policy gaps that affected rural communities. Ultimately, such interventions will be essential for achieving global health goals and improving the well-being of these communities in the long term.

### Supplementary Information

**Additional file 1.** Characteristic of the focus groups.

**Additional file 2.** The interview protocols.

**Additional file 3.** The findings.

## Data Availability

All data used and/or analysed during this study, including the unpublished record are available upon request to the corresponding author.

## Abbreviations

WHO: World Health Organization
ITN: Insecticide-treated bed nets
IRS: Indoor residual spraying
UHC: Universal health coverage
IDIs: In-depth interviews
FGDs: Focus group discussions
Kg: *Kampung*
NRP: National Recovery Plan
UKM: Universiti Kebangsaan Malaysia
PPRT: *Projek Perumahan Rakyat Termiskin*
VCRC: Vector Control Research Centre
CHWs: community health workers
SKPA: *Sukarelawan Penjagaan Kesihatan Asas*
SDG: Sustainable Developmental Goals
