# Supplementary material for: Disentangling the intersection of inequities with health and malaria exposure: key lessons from rural communities in Northern Borneo
Source: Malar J. 2023 Nov 9;22:343. doi: 10.1186/s12936-023-04750-9 (PMC10636872; doi:10.1186/s12936-023-04750-9)
Supplement: Supplementary file 1 — Additional file 1. Characteristic of the focus groups. [file 12936_2023_4750_MOESM1_ESM.docx]

| Focus group characteristic | Name of village (study sites) | Number of participants | Age range (Median) | Gender:  % female  % male | Present individual with history of previous malaria diagnosis (n) | Present individual with household or family members with malaria diagnosis |
| --- | --- | --- | --- | --- | --- | --- |
| Group 1 | Kampung Manduri | 6 | 21 to 55 years old (Median: 32.5) | Female: 6 (100%) | 1 (16.67%) | 100% |
| Group 2 | Kampung Paradason | 6 | 22 to 41 years old (Median: 27.5) | Female: 4 (66.67%)  Male: 2 (33.33%) | 3 (50%) | 100% |
| Group 3 | Kampung Tagumamal Darat | 8 | 27 to 72 years old (Median: 45) | Female: 4 (50%)  Male: 4 (50% | 3 (37.5%) | 100% |
| Group 4 | Kampung Membatu Laut | 6 | 33 to 63 years old (Median: 34.5) | Female: 6 (100%) | 0 (0%) | 100% |
|  |  | 26 | Median age: 34.5 | Female: 20 (76.92%)  Male: 6 (23.08%) | 6 (23.05%) | 100% |
