# Supplementary material for: Disentangling the intersection of inequities with health and malaria exposure: key lessons from rural communities in Northern Borneo
Source: Malar J. 2023 Nov 9;22:343. doi: 10.1186/s12936-023-04750-9 (PMC10636872; doi:10.1186/s12936-023-04750-9)
Supplement: Supplementary file 2 — Additional file 2. The interview protocols. [file 12936_2023_4750_MOESM2_ESM.docx]

**Photovoice Focus Group Protocol**

**Part 1 Opening (10 minutes)**

1.Welcomeing everyone, make introductions and thank participants.

2.Review the purpose of the focus group interview. Inform the aim.

3.Review confidentiality issues, and consent for audio and video recording

4.Review the focus group norms and protocol.

5.Review the photovoice prompt that guides the session. During the session, a review of previous session was done.

**Part 2. Photo-discussion (60 to 90 minutes)**

The acronym “SHOWeD” were used to guide the discussion, through the narration of their chosen photograph.

| **Acronym** | **FFGD moderator prompt** | **Participant’s action** |
| --- | --- | --- |
| **S** | What can you **see** from this photograph? | Participant describes what he or she sees from her/his photograph |
| **H** | How does this relate with you/their life? | Participants describes what is happening in the photograph, and how does it relate with his/her/their life |
| **O** | Why does this photograph relate to **our** inquiry? | Participants describe how the picture relates to her/his/their life |
| **W** | **Why** does these situation/ concern/drivers/barriers exist? | Participants descirbes about the reason as to why the situation/challenges/barriers/drivers exist |
| **D** | What can I/We/They **do** about it? | Participants suggest/recommend/share opinions on concerns and issues raised. |

| **Research aim** | **Research questions** | **Photovoice questions** |
| --- | --- | --- |
| To explore the communities’ perception on malaria | 1.What do you understand about malaria? (What is malaria?  2.What are the factors that influence the malaria preventive behavior of indigenous communities in Sabah, who reside in areas exposed to *P. knowlesi* infection | 1.How can you/your community get infected with monkey malaria?  Prompts  1.When?  2.Where?  3.How?  4.Why?  5.Who?  6. Is there any supernatural beliefs?  7. What misconceptions do people in your community have about malaria?" |
|  | 1.How do the communities avoid malaria/mosquito bites?  2. How do community members perceive the effectiveness of malaria prevention and control interventions implemented by the government or non-governmental organizations? | 1. What are the ways that you/your community do to avoid getting infected with monkey malaria?  2. What are the ways that you/your community do to avoid mosquito bites?  3. How do you or your community avoid mosquito bites?  4.What is your opinion on the current intervention? -bed nets? -spraying? Cream? |
| To explore the communities’ challenges to avoid malaria | 1.What are the challenges that communities face in preventing malaria? -what are the drivers? what are the barriers?  2. How do socioeconomic and cultural factors impact communities' ability to avoid malaria, and what strategies can be developed to address these barriers? | 1.What activities do you/your communities perform during the evening, from 6 pm until 6 am the next day  Prompts  1. Why is it difficult to avoid malaria in your village?  2.Why is it difficult to avoid malaria/mosquito bites during this time period?  3. Why is it easy to avoid malaria/mosquito bites during this period of time?  4. How do cultural beliefs and practices affect the community's attitudes and behaviors towards malaria prevention? |
| To identify their concern and share as new information to policymakers.  Sharing concern on “What you you/we/they do?” | 1.What strategies can be developed to address these barriers? | 1.How can we avoid these challenges to avoid malaria/mosquito bites?  2. How can malaria prevention and control strategies be improved to better meet the needs of the community?  Prompt  1.What can you/the communities do?  2.What can the stakeholders do?  3.What can the government do?  4.How can anyone help this malaria situation in your village? |

**Part 3: Member-checking**

The facilitator identify the meaning from the shared discussion and generate preliminary codes and themes, which were confirmed by the participants.

**Part 4. Conclusion**

The moderator inquire if there is anything else that the participants would like to share before ending the session. Is there anything else you’d like to add before we end the session?

Farewell.

**Final FGD session**

At the final FGD, the moderator invites the participants to reflect on the photovoice process.

1. What is your opinion on photovoice?
2. Did you find it challenging? Prompt with if yes why, if not, why not?
3. Did you enjoy participating in the photovoice study? If yes why, if not, why not?
4. What did you gain throughout the photovoice study?
5. What was it like being part of the photovoice study?
6. Any interesting experience that you would like to share?
7. Any challenging experience that you would like to share?
8. May I contact you again in the future?

**Protocol in-depth interview**

**Part 1 Opening (10 minutes)**

1.Welcoming the community leader, make introductions and thank the participant.

2.Review the purpose of the in-depth interview. Inform the aim.

3.Review confidentiality issues, and consent for audio and video recording

4.Review the interview participant information sheet and consent.

**Part 2. Interview session (60 to 90 minutes)**

| Research aim | Research questions | In depth interview questions |
| --- | --- | --- |
| Communities’ perception on malaria | 1.What do you understand about malaria? (What is malaria?  2.What are the factors that influence the malaria preventive behavior of indigenous communities in Sabah, who reside in areas exposed to *P. knowlesi* infection | 1.How can you/your community get infected with monkey malaria?  Prompts  1.When?  2.Where?  3.How?  4.Why?  5.Who?  6. Is there any supernatural beliefs?  7. What misconceptions do people in your community have about malaria?"  8. What is your opinion on communities’ perception on malaria? |
| To explore the communities’ challenges to avoid malaria | 1.How do the communities avoid malaria/mosquito bites?  2. How do community members perceive the effectiveness of malaria prevention and control interventions implemented by the government or non-governmental organizations? | 1. What are the ways that you/your community do to avoid getting infected with monkey malaria?  2. What are the ways that you/your community do to avoid mosquito bites?  3.What is your opinion on the current intervention? -bed nets? -spraying? Cream? |
| To identify their concern and share as new information to policymakers. | 1.What strategies can be developed to address these barriers? | 1.How can we avoid these challenges to avoid malaria/mosquito bites?  2. How can malaria prevention and control strategies be improved to better meet the needs of the community?  Prompt  1.What can you/the communities do?  2.What can the stakeholders do?  3.What can the government do?  4.How can anyone help this malaria situation in your village? |

**Part 3: Member-checking**

The interviewer identify the meaning from the interview and generate preliminary codes and themes, which were confirmed by the participant.

**Part 4. Conclusion**

The interviewer inquire if there is anything else that the participant would like to share before ending the session. Is there anything else you would like to add before we end the session?

Farewell.
