# Supplementary material for: Disentangling the intersection of inequities with health and malaria exposure: key lessons from rural communities in Northern Borneo
Source: Malar J. 2023 Nov 9;22:343. doi: 10.1186/s12936-023-04750-9 (PMC10636872; doi:10.1186/s12936-023-04750-9)
Supplement: Supplementary file 3 — Additional file 3. The findings. [file 12936_2023_4750_MOESM3_ESM.docx]

| Themes | Subthemes | Findings | Implications | Recommendations |
| --- | --- | --- | --- | --- |
| Challenges in malaria control with limited resources and health disparities in rural communities  *Defined as a condition characterized by difficulties, obstacles or deprivation that communities face in preventing and controlling malaria, due to variety of factors, including inadequate access to essential resources and healthcare services. This challenges can have adverse effects on their health and well-being* | Water access challenges increase malaria risk in rural areas | -Limited access to treated water and inconsistency of water supply increase the risk of malaria  -access to water sources is challenging and surrounded by mosquitoes and larvae  -gravity water system is not effective four houses located far from the main water point. | -Villagers are at high risk of contracting malaria due to limited access to treated water and inconsistent water supply, surrounded by mosquitoes and larvae | The need to improve the social environment to support the health of the population.  -increase the supply of treated water to rural villages, especially those located far from the main water point  -address the mosquito problem by providing electricity and consistent communication signals.  -improve access to healthcare services by building a hospital in the villages or providing transport to the nearest healthcare facility.  -increase the distribution of bed nets to villagers, especially those who cannot afford them, and improve the replacement system for torn bed nets.  -educate villagers about the importance of seeking healthcare services from the formal health sector instead of traditional healers. |
|  | Impact of limited access to electricity on malaria prevention in rural areas | -Lack of electricity causes darkness in houses and contributes to the mosquito presence problem | Lack of electricity contributes to mosquito problem around the house |  |
|  | Unequal internet access in rural areas poses health risks | -Inconsistency of communication signals results in the need for villagers to search for internet hotspots in the forest or plantation areas, exposing them to mosquito bites | Lack of inconsistent communication signals contribute to risk of mosquito bites when individuals seek for areas with available signals |  |
|  | Limited access to healthcare and resources in rural areas hinders malaria control | -The nearest healthcare facility with specialist is located far from the villages, making it challenging for villagers to seek malaria diagnosis and treatment  -Limited resources at the rural clinic delay the distribution of bed nets to villagers, and replacement of torn bed nets is difficult due to affordability issues.  -Majority of community members do not have transport to go to the clinic  -some villagers still seek care from traditional healers for their illnesses as an alternative to the formal health sector. | -Delay in seeking health treatment  -Poor motivation to seek healthcare services  -limited resources at the clinic delay the distribution of bed nets to villagers, and might contribute to seeking treatment for traditional healers |  |
|  | The neglect of community concerns, lack of policies and capacities that hinder malaria control | -Inability to raise their voice on issue that are concerning and important to the communities  Ignorance by certain stakeholders upon the raised issues in the village  Lack of supportive policies  Power imbalances in managing resources | Social disparity in health and other aspects such as economy, health and environment |  |
| The livelihood challenges to malaria prevention due to local socio-economy structures and practices  *Defined as the condition that challenges communities whose livelihood are dependent on outdoor activities which exposes them to malaria* | - | -livelihood challenges and malaria risk due to the dependence of outdoor activities that exposes them to malaria  -limitation of adequate malaria prevention methods for outdoor usage  -quality of housing in the village can affect their risk of malaria  -villagers often prioritised income generation over the need to prevent malaria, given the need to support their families |  | -Innovation in malaria control  -Collaboration in research and malaria program  -the need for alternative mosquito control products that can be used during outdoors  -Participants highlight the need of government support in providing adequate housing and other resources that can reduce their risk of malaria |
| Environmental degradation and malaria risk in rural communities  *Defined as the environmental changes which resulted in negative impacts on the environment and the health of the villagers* |  | -communities live where forests, plantation, and farms are part of their everyday life  -there are fewer natural forests as many of the lands were changed into to plantation and farming areas  -the presence of monkeys in and around the villages predisposed a higher risk of “monkey malaria”  -planting fruit trees or oil palms near the village can increase the risk of “monkey malaria”  -no proper roads created temporary water pools suitable for mosquito to breed | - the environmental changes in the villages have led to an increase in the risk of monkey malaria, and the lack of proper infrastructure exacerbates the problem  -community leaders are concerned about the future of their villages that put communities to negative health outcomes, considering the given impact of anthropogenic activities on the communities  -present lack of community involvement in anthropogenic projects around the village | -involve the community in decision-making process for development of projects to ensure their concerns and needs are considered  -avoid planting fruit trees or oil palms near the village to decrease the risk of monkey malaria  -build proper roads and infrastructure in the villages to reduce the prevalence of mosquito breeding areas  -promote reforestation and sustainable land use practises to help mitigate the negative effects of deforestation  -raise awareness about the impact of environmental degradation on public health and the economy. |
